# Medium-term outcome of recipients of marginal donor hearts selected with new stress-echocardiographic techniques over standard criteria

**DOI:** 10.1186/1476-7120-12-20

**Published:** 2014-06-16

**Authors:** Tonino Bombardini, Giorgio Arpesella, Massimo Maccherini, Francesco Procaccio, Luciano Potena, Sonia Bernazzali, Ornella Leone, Eugenio Picano

**Affiliations:** 1 Institute of Clinical Physiology, National Research Council, Via Moruzzi 1, 56124 Pisa, Italy; 2 Cardiac Surgery, Heart and Lung Transplantation Program, University of Bologna and S. Orsola-Malpighi Hospital, Bologna, Italy; 3 Heart Transplantation Division, Department of Cardiac Surgery, University of Siena, Siena, Italy; 4 Intensive Care Coordination, National Transplant Centre, Italian National Institute of Health, Rome, Italy; 5 Neuro Intensive Care Unit, University City Hospital, Verona, Italy; 6 Cardiovascular Department, University of Bologna and S. Orsola-Malpighi Hospital, Bologna, Italy; 7 Department of Pathology, University of Bologna and S. Orsola-Malpighi Hospital, Bologna, Italy

**Keywords:** Heart transplant, Heart donor shortage, Stress echocardiography, Reversible wall motion abnormalities, Hormonal treatment

## Abstract

**Background:**

Heart transplantation is limited by severe donor organ shortage. Regardless of the changes made in the acceptance of marginal donors, any such mechanism cannot be considered successful unless recipient graft survival rates remain acceptable. A stress echo-driven selection of donors has proven successful in older donors with normal left ventricular resting function and in standard donors with reversible resting left ventricular dysfunction acutely improving during stress, or slowly improving (over hours) during intensive hormonal treatment. Aim of this study is to assess the medium-term outcome of recipients of marginal donor hearts selected with new echocardiographic techniques over standard criteria.

**Methods and results:**

We enrolled 43 recipients of marginal donor hearts: age > 55 years, or < 55 years but with concomitant risk factors, n = 32; acutely improving during stress, n = 3; or slowly improving during hormonal treatment, n = 8. At follow-up (median, 30 months; interquartile range, 21–52 months), 37 of the recipients were still alive. One-year survival was 93%.

**Conclusion:**

The strict use of new stress-echocardiographic techniques over standard criteria of marginal donor management, together with comprehensive monitoring of the donor, has the potential to substantially increase the number of donor hearts without adverse effects on recipient medium-term outcome.

## Introduction

Heart transplantation is an established procedure in end-stage heart failure patients, albeit limited by severe and incremental donor organ shortage. In Europe every year a pool of ≈ 4500 unused hearts (500 in Italy) with permission granted for heart donation is estimated, from which additional transplants could be generated, with more confidence in their post-transplantation performance (Council of Europe, Donation and Transplantation, 2011) [1,2]. A stress echo-driven selection of donors has proven successful in three settings: 1) older donors with normal left ventricular (LV) resting function and negative stress echo [3,4]; 2) reversible resting left ventricular dysfunction acutely improving over minutes during stress [5]; 3) regional and global LV dysfunction slowly improving (over hours) during intensive hormonal treatment (HT) [6-11]. In all three conditions, encouraging results and short-term progress have been reported in preliminary proof of principle studies, but data on medium-term outcome have been conspicuously lacking to date. Aim of this study is to assess the medium-term outcome of recipients of marginal donor hearts selected via new echocardiographic techniques over standard criteria.

## Methods

**Figure 1:**
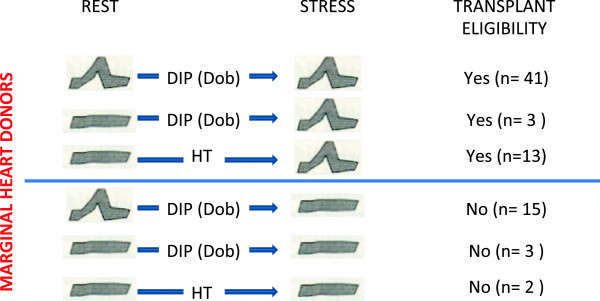
**Donor heart eligibility criteria.** Upper panel. Eligible heart: non-inducible ischemia (first row) in hearts with normal rest LV function; viability response at stress echo (second row) or at HT (third row) in stunned hearts with abnormal rest LV function. Lower panel. Non-eligible heart: stress echo positivity (first row) in hearts with normal rest LV function, excluding the heart from transplant; lack of viability response at stress echo (second row) or at HT (third row) in hearts with abnormal rest LV function.

According to a methodology previously described in detail, in this analysis we enrolled three different categories of potential marginal donors: 1) 97 patients enrolled in the Adonhers project [3,4,12], consisting of potential donors aged > 55 years, or < 55 years but with concomitant risk factors; 2) 6 subjects with resting wall motion abnormality undergoing pharmacological stress echo [5]; 3) 15 subjects with hemodynamic instability [13] Table 1. In all cases, LV wall motion score index (WMSI) was assessed and graded on a scale from 1 (normal) to 4 (dyskinetic) in each of the 17 segments at rest and following intervention (pharmacological stress or HT) [14,15]. Ejection fraction was calculated using the biplane Simpson rule [16] and LV elastance as the ratio of systolic pressure by cuff sphygmomanometry to LV end-systolic volume [17]. The intervention consisted of dipyridamole infusion (0.84 mg/kg over 6′, n = 59) or dobutamine (up to 40 mcg/kg/min, n = 4) or – for HT – in infusion with insulin, methylprednisolone, vasopressin and T3. Heart eligibility criteria have been previously described in detail [3-5,13] and are schematically summarized in Figure 1. Briefly, in presence of normal resting function, the eligible heart showed normal regional and global wall motion; in presence of abnormal resting function, the eligible heart showed regional and global wall motion restoring over minutes with pharmacological stress or over days with HT.

**Table 1:** Eligibility criteria in marginal donors by echocardiographic techniques

	**Donors aged > 55 years or with ≥ 3 risk factors**	**Donors with rest wall motion abnormalities**	**Donors with hemodynamic instability/LV dysfunction**
N. initially recruited	97	6	15
N. potentially eligible studied patients	57	6	15
Intervention (stress)	DIP (dob)	DIP (dob)	HT
Echo assessment following intervention	Minutes	Minutes	Hours
WMSI rest	1	> 1	1/> 1
WMSI peak	1	1	
LVEF% rest	59 ± 10	53 ± 8	48 ± 14
LVEF% peak	67 ± 9	58 ± 7	59 ± 3
Contractile reserve	+	+	+
Viability	NA	+	+
Stress dismissed	N = 15	N = 3	
Eligible non-transplanted	N = 9	-	N = 7
Eligible transplanted hearts	N = 32	N = 3	N = 8
Donor characteristics of transplanted hearts			
Age (years)	55 ± 7	34 ± 13	50 ± 9
Male gender	53 (58%)	3 (50%)	8 (53%)
BSA (m^2^)	1.81 ± 0.19	1.95 ± 0.14	1.90 ± 0.19
*Cause of death*			
Anoxia	1	-	-
Cerebrovascular accident	22	1	7
Head trauma	9	2	1
Troponin > 0.14 micrograms/L	21	3	8

BSA = Body Surface Area; DIP = Dipyridamole; DOB = Dobutamine; WMSI = Wall Motion Score Index.

The pts in the Ht protocol were considered eligible only after coronary angiography ruled out the presence of coronary stenosis.

### Ethical committee

The ethics committee of the Emilia-Romagna and Tuscany regions of Italy approved the Aged Donor Heart Rescue by Stress Echo Project in 2004 (number 142/2004/U/Oss, October 19, 2004). Partial funding for the Stress Echo project was provided by the Italian Health Ministry (CCM project #48, 2010). On May 11, 2011 the Italian National Transplant Center/Italian National Institute of Health (CNT/ISS 2011) approved the Guidelines: Increase available organs for heart transplant with heart assessed by stress echocardiography in older donors or in donors with several risk factors, with a second-opinion telemedicine system from the core echo lab, IFC-Pisa.

### Coronary angiography

Donors enrolled in the stress echo protocols were enrolled in neurological intensive care units, without direct access to coronary angiography facilities. Standard coronary angiography with IVUS was performed 1 month after heart TX [18]. Donors enrolled in the HT protocol underwent coronary angiography before heart harvesting. Once transplanted, all patients underwent usual surveillance and immunosuppression protocol and infection prophylaxis, with serial endomyocardial biopsies according to guidelines [19].

### Statistical analysis

SPSS version 21 for Windows (SPSS, Inc., Chicago, IL, USA) was used for statistical analyses. The statistical analyses included descriptive statistics (frequencies and percentages for categorical variables and mean ± SD for continuous variables). Patient’s survival curves were estimated using the Kaplan–Meier method and compared between groups using the log-rank test. The survival was censored at the time of death. For patients who were still alive, survival was censored at date of last known follow-up. Two-tailed p < 0.05 was considered statistically significant.

## Results

**Figure 2:**
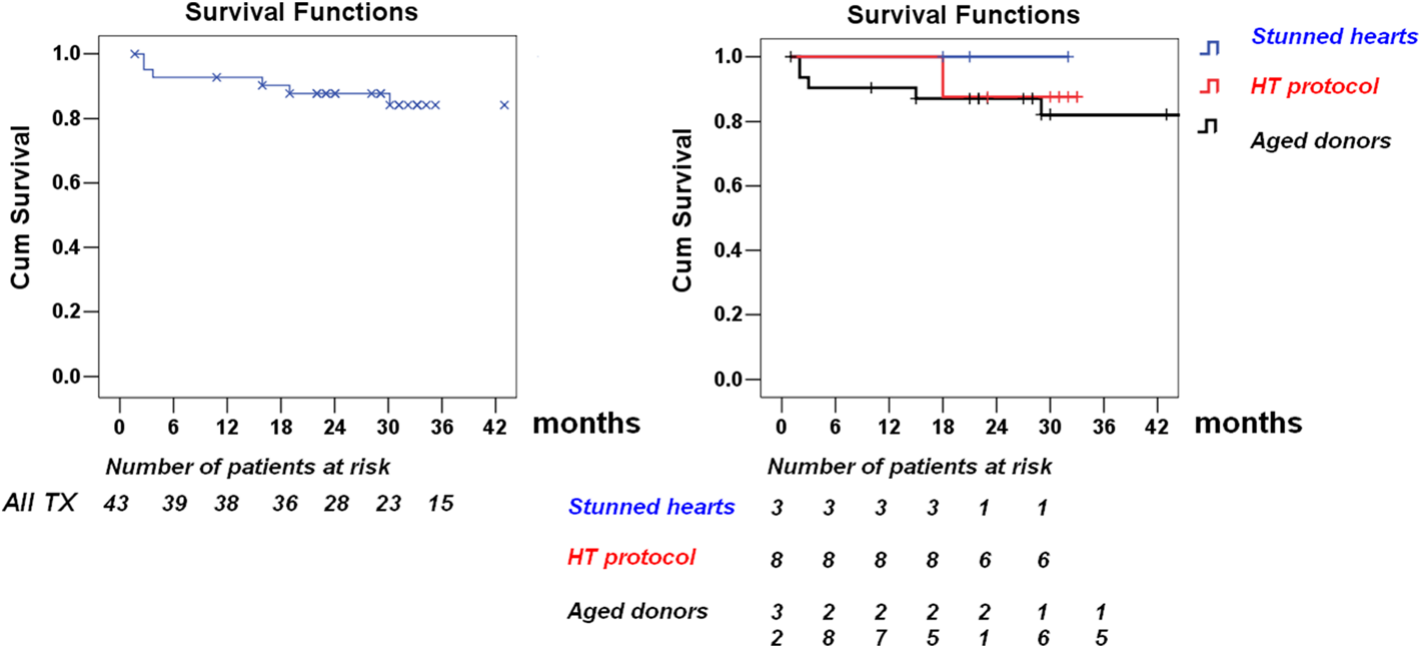
**Survival curves in recipients of donor hearts selected with new echocardiographic techniques.** Right panel. The survival curves of the 3 groups of pts. Left panel. The survival curve of the whole group of patients.

From April 2006 to April 2013, of the initial set of 118 enrolled subjects, 44 were deemed eligible after stress echocardiography, and 15 after HT. Nine patients eligible by stress echo were not transplanted due to: LV hypertrophy (N = 2 cases), epicardial coronary calcium at surgical inspection (N = 2), malignancy (N = 1), HCV positivity (N = 1), lack of a matching recipient (N = 3). Seven eligible donors enrolled in the HT protocol were not transplanted due to: opposition (N = 3), tubercolosis (N = 1), malignancy (N = 1), coronary artery stenosis at pre-harvesting angiography (N = 2); 43 marginal donor hearts were eventually transplanted Table 2. The recipients were predominantly male (33 out of 43), with a mean age of 56 ± 9 years. Patients were enrolled from a waiting list: 5 recipients were United Network for Organ Sharing (UNOS) status 1A (with mechanical circulation support devices as bridging for HT), 7 recipients were status 1B, and 31 were status 2 [20]. One recipient with systemic amyloidosis received simultaneous liver Tx, one with chronic dialysis treatment received simultaneous kidney TX. Six recipients died at follow-up: two recipients had primary graft failure after HT, one recipient with severe pre-TX pulmonary hypertension and one recipient with previously implanted VAD as bridge to TX; two died (at 2 months and at 18 months) from general sepsis; one died at 32 months from allograft vasculopathy [21] in recurrent multiple myeloma; one died at 16 months from newly diagnosed liver cancer. Two of the **43** eligible transplanted hearts showed significant (70%) stenosis of a major coronary vessel (LAD in one, RCA in one) on 1-month post-HT coronary angiography, and underwent PCI with stenting. At follow-up (median, 30 months; interquartile range, 21–52 months), 37 of the recipients were still alive (Figure 2). One-year survival was 93%. Verification by autopsy in eligible hearts not transplanted showed absence of significant abnormalities (coronary artery disease or cardiomyopathy).

**Table 2:** Recipients of donors selected by echocardiographic techniques

	**Donors aged > 55 years or with ≥ 3 risk factors**	**Donors with rest wall motion abnormalities**	**Donors with hemodynamic instability/LV dysfunction**
Eligible transplanted hearts	N = 32	N = 3	N = 8
Cold ischemia time (minutes)	178 ± 26	161 ± 16	150 ± 19
*Recipient characteristics*			
Age (years)	56 ± 9	53 ± 4	56 ± 7
Male gender	26	3	4
*UNOS state*			
*1A*	2		3
*1B*	7		
*2*	23	3	5
*Recipient disease*			
DCM	9		3
DC CHD	12	1	3
DC Valvular	2		1
HCM	4	1	
Restrictive CMP in amyloidosis	3		1
DC other	2	1	
TX associated to heart TX	2 (Liver N = 1, Kidney N = 1)		
Survivors N	27	3	7
FOLLOW-UP, months	44 ± 24	24 ± 7	31 ± 4
Death, N	5	-	1
*Deaths (cause, post TX months)*			
PGF	2 (2 months)		
Sepsis	1 (2 months)		1 (18 months)
Cancer	1 (16 months)		
Recurrent systemic myeloma	1 (32 months)		

CHD = Coronary heart disease; CMP = Cardiomyopathy; DC = Dilated cardiomyopathy; DCM = Idiopathic dilated cardiomyopathy; HCM = Hypertrophic cardiomyopathy; PGF = Primary graft failure; TX = Heart transplant.

## Discussion

This study shows that the surgical mortality rates of adults who underwent heart transplantation of marginal donors selected by means of new echocardiographic techniques are excellent, and that medium-term survival is acceptable. The most common cause of death within 1 year is graft failure, followed by infection. Notably, only one of our patients died due to cardiac allograft vasculopathy, in recurrent multiple myeloma. Additional studies are needed to assess the impact of cardiac allograft vasculopathy in marginal donors.

### Stress echocardiography to select marginal donor hearts: strengths and weaknesses

Supply of donor hearts is a critical rate-limiting step in heart transplantation. An effective way to solve the current shortage would be to accept an upward shift of the age cut-off limit and to accept for transplant normal but temporarily stunned donor hearts [4,5,13,22]. Pharmacological stress echo (with stress being classical pharmacological stress or HT extended for several hours) can identify aged good hearts with normal resting function (and normal response during stress) and even with abnormal resting function (and functional recovery during stress or following HT) [23,24].

The suggested first choice was dipyridamole, with dobutamine as the alternative, acceptable second choice. There were two reasons for the choice: dipyridamole is equally as accurate but technically simpler than dobutamine because of a lesser increase in heart rate, and the image quality is therefore less degraded during stress [3,4] (this aspect is especially important in the technically challenging theater of testing potential donors who have high resting heart rates), and further catecholamine stress with dobutamine is in principle undesirable for these patients, because they already have high, and potentially toxic, levels of circulating catecholamines, which may damage the heart [19,22,25].

Although appealingly simple, a stress-echo driven approach depends on the qualitative, operator-dependent assessment of regional wall motion, requiring strict criteria in the process of execution, acquisition, analysis and interpretation. The Italian National Transplant Center recently approved the stress echo methodology for selection of older donor hearts (CNT/ISS; 2011) for all Italian regions [25,26]. The Core Echo Lab, Pisa (IFC, National Research Council) is responsible for accreditation of cardiologists to carry out stress echo in each center involved in the project, certification of a “second opinion” in near real time, and final acceptance of the donation [12]. For this, an Italian network of stress echo laboratories has been organized by IFC (CNR, Pisa). All network laboratories have agreed to pass a quality control examination on stress echo reading before entering the study, to code the LV segments similarly, and to adopt a common scoring system for wall motion analysis. The Transplantation Network involves the cardiac-transplant program throughout Italy. There are no specific requirements for instruments and there are no additional costs for local health agencies. In this way, at nearly no extra cost, the research infrastructure allows access to donation to feed an activity such as heart transplantation with high running fixed costs and obvious beneficial impact on the sustainability of the health system. With standard Italian health care costs in the public system, a stress echo is priced at around € 100, and a heart transplant (made possible by stress echo-driven selection) around € 80,000.

### Limitations of the study

In theory, it may seem more convenient to perform stress echocardiography at the bedside rather than transport the donor to the cardiac catheterization laboratory [25-29]. The concept of replacing an invasive test requiring transportation to the cardiac catheterization laboratory with one that could potentially be performed at the bedside is appealing; however, the practicality of bedside stress echocardiography requires further evaluation [25]. The decision to screen more marginal donors noninvasively will increase the risk for hearts with CAD being accepted for transplantation. The implications of this are unclear. It has been demonstrated that the presence of donor-transmitted coronary atherosclerosis does not accelerate the progression of intimal hyperplasia or affect the 3-year prognosis of transplant recipients. Recent studies indicate that deleterious transplant vasculopathy (TVP) as a result of chronic rejection is multifactorial and that atherosclerotic plaque in the donor heart may not necessarily progress to TVP [30-32]. Instead, using serial Intravascular Ultrasound (IVUS) measurements, Li et al. [33] demonstrated that pre-existing donor atherosclerotic lesions do not accelerate the development of TVP either at the site of pre-existing donor atherosclerosis or elsewhere within the same artery. However, donor-transmitted coronary atherosclerosis increases the incidence of cardiac allograft vasculopathy. Recently, Grauhan et al. [18] described an overall prevalence of donor-transmitted coronary atherosclerosis of 7.0%, and he stated that donor screening without coronary angiogram overlooks a significant proportion of coronary lesions. In that study, the prevalence of donor transmitted CAD in recipients who underwent coronary angiography within 6 months post-transplantation was 5.2%, whereas it was 15.1% on autopsy in those recipients who died within 6 months without coronary angiogram. Among all patients with early graft failure, prevalence was as high as 22.8% indicating that donor CAD represents a significant risk factor for early graft failure [34].

## Conclusions

The medium-term outcome of recipients of marginal donor hearts selected with new echocardiographic techniques over standard criteria demonstrated survival rates similar to that of recipients of “standard” donor hearts. As waiting lists for heart transplantation continue to grow, continuous changes in practice patterns of donor heart usage are most urgent. It is believed that about 15,000 patients would potentially benefit from a heart transplant, if the acceptance criteria included ‘marginal’ donors up to 55 years of age, and about 40,000-70,000 patients would benefit, if the acceptance age was extended to 65 years [35]. In “younger marginal donors”, aggressive assessment and optimal management of donor left ventricular dysfunction offer tremendous potential for increasing cardiac donor utilization since a significant proportion of hearts are declined for reasons of ‘poor ventricular function’. Strong evidence indicates that grafts from younger donors with left ventricular dysfunction can completely recover to normal function over time in the donor [36] and following transplantation into a recipient [37]. Although echocardiography is very effective in screening for anatomical (especially valvular) anomalies of the heart, use of a single echo examination in terms of a ‘snapshot assessment’ of pump function to determine the physiological suitability of a donor graft is not well-supported by evidence [24]. However, regardless of changes in the acceptance of marginal donors, any such mechanism will not be considered successful unless recipient graft survival rates in center-specific outcome analyses remain acceptable. The strict use of new stress-echocardiographic techniques over standard criteria of marginal donor management, together with comprehensive monitoring of the donor, has been shown to have the potential to increase substantially the number of donor hearts without adverse effects on recipient medium-term outcome.

## Abbreviations

ADONHERS: Aged donor heart rescue by stress echo project; CNT: Italian National Transplant Center; HCV: Hepatitis c virus; HT: Hormonal treatment; ISS: Italian National Institute of Health; LAD: Left anterior descending artery; LV: Left ventricular; PCI: Percutaneous coronary intervention; UNOS: United network for organ sharing; RCA: Right coronary artery; TX: Transplant; VAD: Ventricular assist device; WMSI: Wall motion score index.

## Competing interests

The authors of this manuscript have no conflicts of interest to disclose.

## Authors’ contributions

TB, GA and EP conceived this study, performed the data analysis, and drafted the manuscript; MM, FP and OL gave a contribution to preparation of study design, data discussion, and critical revision of the manuscript; LP and SB were responsible for data collection and revised the manuscript. All authors read and approved the final manuscript.

## References

[B1] Domínguez-Gil B Haase-Kromwijk B Van Leiden H Neuberger J Coene L Morel P Corinne A Muehlbacher F Brezovsky P Costa AN Rozental R Matesanz R European Committee (Partial Agreement) on Organ Transplantation. Council of Europe (CD-P-TO) Current situation of donation after circulatory death in European countries Transpl Int 2011 24 676 686 10.1111/j.1432-2277.2011.01257.x 21504489

[B2] Dronavalli VB Banner NR Bonser RS Assessment of the potential heart donor: a role for biomarkers? J Am Coll Cardiol 2010 56 352 361 10.1016/j.jacc.2010.02.055 20650355

[B3] Leone O Gherardi S Targa L Pasanisi E Mikus P Tanganelli P Maccherini M Arpesella G Picano E Bombardini T Stress echocardiography as a gatekeeper to donation in aged marginal donor hearts: anatomic and pathologic correlations of abnormal stress echocardiography results J Heart Lung Transplant 2009 28 1141 9 10.1016/j.healun.2009.05.029 19782600

[B4] Bombardini T Gherardi S Arpesella G Maccherini M Serra W Magnani G Del Bene R Picano E Favorable short-term outcome of transplanted hearts selected from marginal donors by pharmacological stress echocardiography J Am Soc Echocardiogr 2011 24 353 62 10.1016/j.echo.2010.11.014 21440213

[B5] Bombardini T Gherardi S Leone O Sicari R Picano E Transplant of stunned donor hearts rescued by pharmacological stress echocardiography: a “proof of concept” report Cardiovasc Ultrasound 2013 11 27 10.1186/1476-7120-11-27 23915276 PMC3735394

[B6] Wood KE Becker BN McCartney JG D’Alessandro AM Coursin DB Care of the potential organ donor N Engl J Med 2004 351 2730 2739 10.1056/NEJMra013103 15616207

[B7] Novitzky D Cooper DKC Rosendale JD Kauffman HM Hormonal therapy of the brain-dead organ donor: experimental and clinical studies Transplantation 2006 82 1396 1401 10.1097/01.tp.0000237195.12342.f1 17164704

[B8] Zaroff JG Babcock WD Shiboski SC Solinger LL Rosengard BR Temporal changes in left ventricular systolic function in heart donors: results of serial echocardiography J Heart Lung Transplant 2003 22 383 388 10.1016/S1053-2498(02)00561-2 12681416

[B9] Taniguchi S Kitamura S Kawachi K Doi Y Aoyama N Effects of hormonal supplements on the maintenance of cardiac function in potential donor patients after cerebral death Eur J Cardio-thorac Surg 1992 6 96 102 10.1016/1010-7940(92)90082-9 1581088

[B10] Wheeldon DR Potter CD Oduro A Wallwork J Large SR Transforming the “unacceptable” donor: outcomes from the adoption of a standardized donor management technique J Heart Lung Transplant 1995 14 734 742 7578183

[B11] Mcdonald PS Aneman A Bhonagiri D Jones D O’Callaghan G Silvester W Watson A Dobb G A systematic review and meta-analysis of clinical trials of thyroid hormone administration to brain dead potential organ donors Crit Care Med 2012 40 1635 1644 10.1097/CCM.0b013e3182416ee7 22511141

[B12] Franchi D Cini D Arpesella G Gherardi S Calamai I Barletta G Valente S Pasanisi E Sansoni S Ricci C Serra W Picano E Bombardini T Second-opinion stress tele-echocardiography for the Adonhers (Aged donor heart rescue by stress echo) project Cardiovasc Ultrasound 2010 8 20 10.1186/1476-7120-8-20 20515476 PMC2887413

[B13] Casartelli M Bombardini T Simion D Gaspari MG Procaccio F Wait, treat and see: echocardiographic monitoring of brain-dead potential donors with stunned heart Cardiovasc Ultrasound 2012 10 25 10.1186/1476-7120-10-25 22721412 PMC3439356

[B14] Pellikka PA Nagueh SF Elhendy AA Kuehl CA Sawada SG American Society of Echocardiography American Society of Echocardiography recommendations for performance, interpretation, and application of stress echocardiography J Am Soc Echocardiogr 2007 20 1021 41 10.1016/j.echo.2007.07.003 17765820

[B15] Sicari R Nihoyannopoulos P Evangelista A Kasprzak J Lancellotti P Poldermans D Voigt JU Zamorano JL European Association of Echocardiography Stress echocardiography expert consensus statement—executive summary: European Association of Echocardiography (EAE) (a registered branch of the ESC) Eur Heart J 2009 30 278 89 19001473 10.1093/eurheartj/ehn492

[B16] Gardin JM Adams DB Douglas PS Feigenbaum H Forst DH Fraser AG Grayburn PA Katz AS Keller AM Kerber RE Khandheria BK Klein AL Lang RM Pierard LA Quinones MA Schnittger I American Society of Echocardiography Recommendations for a standardized report for adult transthoracic echocardiography: a report from the American Society of Echocardiography’s Nomenclature and Standards Committee and Task Force for a Standardized Echocardiography Report J Am Soc Echocardiogr 2002 15 275 90 10.1067/mje.2002.121536 11875394

[B17] Bombardini T Correia MJ Cicerone C Agricola E Ripoli A Picano E Force frequency relationship in the echocardiography laboratory: a noninvasive assessment of Bowditch treppe J Am Soc Echocardiogr 2003 16 646 55 10.1016/S0894-7317(03)00221-9 12778025

[B18] Grauhan O Patzurek J Hummel M Lehmkuhl H Dandel M Pasic M Weng Y Hetzer R Donor-transmitted coronary atherosclerosis J Heart Lung Transplant 2003 22 568 73 10.1016/S1053-2498(02)00655-1 12742420

[B19] Costanzo MR Dipchand A Starling R Anderson A Chan M Desai S Fedson S Fisher P Gonzales-Stawinski G Martinelli L McGiffin D Smith J Taylor D Meiser B Webber S Baran D Carboni M Dengler T Feldman D Frigerio M Kfoury A Kim D Kobashigawa J Shullo M Stehlik J Teuteberg J Uber P Zuckermann A Hunt S Burch M The International Society for Heart and Lung Transplantation guidelines for the care of heart transplant recipients J Heart Lung Transplant 2010 29 914 56 10.1016/j.healun.2010.05.034 20643330

[B20] Osaki S Edwards NM Johnson MR Velez M Munoz A Lozonschi L Murray MA Proebstle AK Kohmoto T Improved survival after heart transplantation in patients with bridge to transplant in the recent era: a 17-year single-center experience J Heart Lung Transplant 2009 28 591 7 10.1016/j.healun.2009.03.008 19481020

[B21] Mehra MR Contemporary concepts in prevention and treatment of cardiac allograft vasculopathy Am J Transplant 2006 6 1248 1256 10.1111/j.1600-6143.2006.01314.x 16686747

[B22] Nguyen H Zaroff JG Neurogenic stunned myocardium Curr Neurol Neurosci Rep 2009 6 486 491 19818236 10.1007/s11910-009-0071-0

[B23] Lietz K John R Mancini DM Edwards NM Outcomes in cardiac transplant recipients using allografts from older donors versus mortality on the transplant waiting list; implications for donor selection criteria J Am Coll Cardiol 2004 43 1553 1561 10.1016/j.jacc.2004.02.002 15120811

[B24] Zaroff JG Rosengard BR Armstrong WF Babcock WD D’Alessandro A Dec GW Edwards NM Higgins RS Jeevanandum V Kauffman M Kirklin JK Large SR Marelli D Peterson TS Ring WS Robbins RC Russell SD Taylor DO Van Bakel A Wallwork J Young JB Consensus conference report: maximizing use of organs received from cadaver donor-cardiac recommendations, March 28–29, 2001, Crystal City, VA Circulation 2002 106 836 841 10.1161/01.CIR.0000025587.40373.75 12176957

[B25] Fine NM Pellikka PA Pharmacologic stress echocardiography for the assessment of organ suitability for heart transplantation: casting a broader net in search of donors J Am Soc Echocardiogr 2011 24 363 6 10.1016/j.echo.2011.02.006 21440214

[B26] Cullen MW Pellikka PA Recent advances in stress echocardiography Curr Opin Cardiol 2011 26 379 384 10.1097/HCO.0b013e328349035b 21730830

[B27] Bombardini T Gherardi S Marraccini P Schlueter MC Sicari R Picano E The incremental diagnostic value of coronary flow reserve and left ventricular elastance during high-dose dipyridamole stress echocardiography in patients with normal wall motion at rest Int J Cardiol 2013 168 1683 1684 10.1016/j.ijcard.2013.03.076 23601214

[B28] Piérard LA De Landsheere CM Berthe C Rigo P Kulbertus H Identification of viable myocardium by echocardiography during dobutamine infusion in patients with myocardial infarction after thrombolytic therapy: comparison with positron emission tomography J Am Coll Cardiol 1990 15 1021 1031 10.1016/0735-1097(90)90236-I 2312956

[B29] Picano E Stress echocardiography. From pathophysiological toy to diagnostic tool Circulation 1992 85 1604 1612 10.1161/01.CIR.85.4.1604 1555297

[B30] Pethig K Heublein B Kutschka I Haverich A Systemic inflammatory response in cardiac allograft vasculopathy: high sensitive C-reactive protein is associated with progressive luminal obstruction Circulation 2000 102 19 Suppl 3 III233 236 11082393 10.1161/01.cir.102.suppl_3.iii-233

[B31] Pethig K Klauss V Heublein B Mudra H Westphal A Weber C Theisen K Haverich A Progression of cardiac allograft vascular disease is assessed by serial intravascular ultrasound: a correlation to immunological and non-immunological risk factors Heart 2000 84 494 498 10.1136/heart.84.5.494 11040007 PMC1729477

[B32] Wittwer T Pethig K Heublein B Franke U Haverich A Wahlers T Cardiac allograft vasculopathy in heart transplant recipients: a bacteriosclerosis by Chlamydia pneumonia? J Heart Lung Transplant 2001 20 196 11250340 10.1016/s1053-2498(00)00412-5

[B33] Li H Tanaka K Anzai H Oeser B Lai D Kobashigawa JA Tobis JM Influence of pre-existing donor atherosclerosis on the development of cardiac allograft vasculopathy and outcomes in heart transplant recipients J Am Coll Cardiol 2006 47 2470 2476 10.1016/j.jacc.2006.01.072 16781375

[B34] Uretsky BF Kormos RL Zerbe TR Lee A Tokarczyk TR Murali S Reddy PS Denys BG Griffith BP Hardesty RL Armitage JM Arena VC Cardiac events after heart transplantation: incidence and predictive value of coronary arteriography J Heart Lung Transplant 1992 11 3 Pt 2 S45 51 1622998

[B35] Massad MG Current trends in heart transplantation Cardiology 2004 101 79 10.1159/000075988 14988629

[B36] Kono T Nishina T Morita H Hirota Y Kawamura K Fujiwara A Usefulness of lowdose dobutamine stress echocardiography for evaluating reversibility of brain death-induced myocardial dysfunction Am J Cardiol 1999 84 578 82 10.1016/S0002-9149(99)00382-3 10482159

[B37] Jeevanandam V Furukawa S Prendergast TW Todd BA Eisen HJ McClurken JB Standard criteria for an acceptable donor heart are restricting heart transplantation Ann Thorac Surg 1996 62 1268 75 10.1016/0003-4975(96)00626-1 8893556

